# Evaluation of Cumulative Meta-analysis of Rare Events as a Tool for Clinical Trials Safety Monitoring

**DOI:** 10.1001/jamanetworkopen.2020.15031

**Published:** 2020-09-04

**Authors:** George C. M. Siontis, Adriani Nikolakopoulou, Orestis Efthimiou, Lorenz Räber, Stephan Windecker, Peter Jüni

**Affiliations:** 1 Department of Cardiology, Bern University Hospital, University of Bern, Bern, Switzerland; 2 Institute of Social and Preventive Medicine, University of Bern, Bern, Switzerland; 3 Applied Health Research Centre, Li Ka Shing Knowledge Institute of St Michael's Hospital, Toronto, Ontario, Canada; 4 Department of Medicine and Institute of Health Policy, Management and Evaluation, University of Toronto, Toronto, Ontario, Canada

## Abstract

This meta-analysis evaluates the use of cumulative meta-analysis of rare events as a tool for safety monitoring of clinical trials using the example of coronary bioresorbable vascular scaffold–associated thrombosis.

## Introduction

The continued vigilance in safety monitoring in randomized clinical trials (RCTs) is critical as more data and experience are accumulated.^1^ Emerging safety profiles of therapeutic interventions during longer follow-up may cast doubt on earlier conclusions about benefit-risk assessment.^1,2^ Along this line, cumulative meta-analysis has been proposed as a tool to evaluate evidence aggregation. We retrospectively assessed how cumulative meta-analysis could serve as a safety monitoring tool to identify the time point when firm evidence for safety concerns of a rare outcome becomes available.

## Methods

For this meta-analysis, we assessed the withdrawn polymeric everolimus-eluting coronary bioresorbable vascular scaffold (BVS) (Absorb; Abbott Vascular). The BVS received CE mark approval in January 2011 and US Food and Drug Administration approval in July 2016. In September 2017, the manufacturer voluntarily withdrew the device owing to safety concerns (increased risk of scaffold-related thrombosis) after it had been available for clinical use for more than 6 years in Europe and 1 year in the US. This study followed the Preferred Reporting Items for Systematic Reviews and Meta-analyses (PRISMA) reporting guideline.

We retrieved all available reports of RCTs comparing the BVS with metallic everolimus-eluting stents for percutaneous coronary interventions by searching PubMed, CENTRAL, and websites of major cardiology meetings occurring before May 31, 2019. Device-related (scaffold or stent) definite or probable thrombosis was the safety outcome of interest. We used Mantel-Haenszel (fixed-effects model) cumulative meta-analysis to summarize accumulated rare events over time and computed odds ratios (ORs) at each time point. All *P* values are 2-sided, and *P* < .05 was considered statistically significant. Analyses were performed using R, version 3.3.2 (The R Foundation for Statistical Computing).

## Results

**Figure 1.**
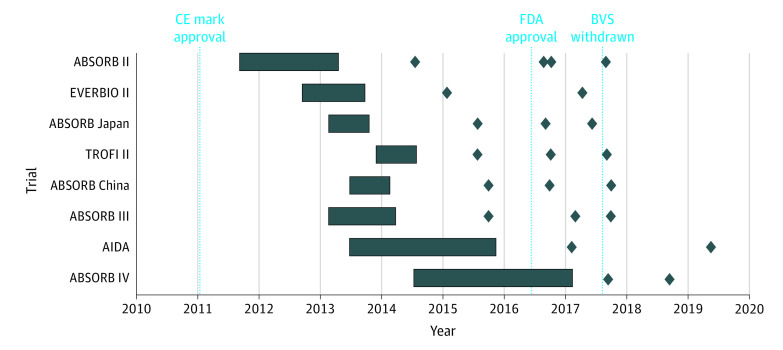
Recruitment Periods Across Trials Comparing the Absorb Polymeric Everolimus-Eluting Bioresorbable Vascular Scaffold With the Metallic Everolimus-Eluting Stent The horizontal blue boxes indicate the recruitment period for each individual trial. Diamonds correspond to the publication of follow-up data for each study over time. BVS indicates bioresorbable vascular scaffolds; FDA, US Food and Drug Administration.

**Figure 2.**
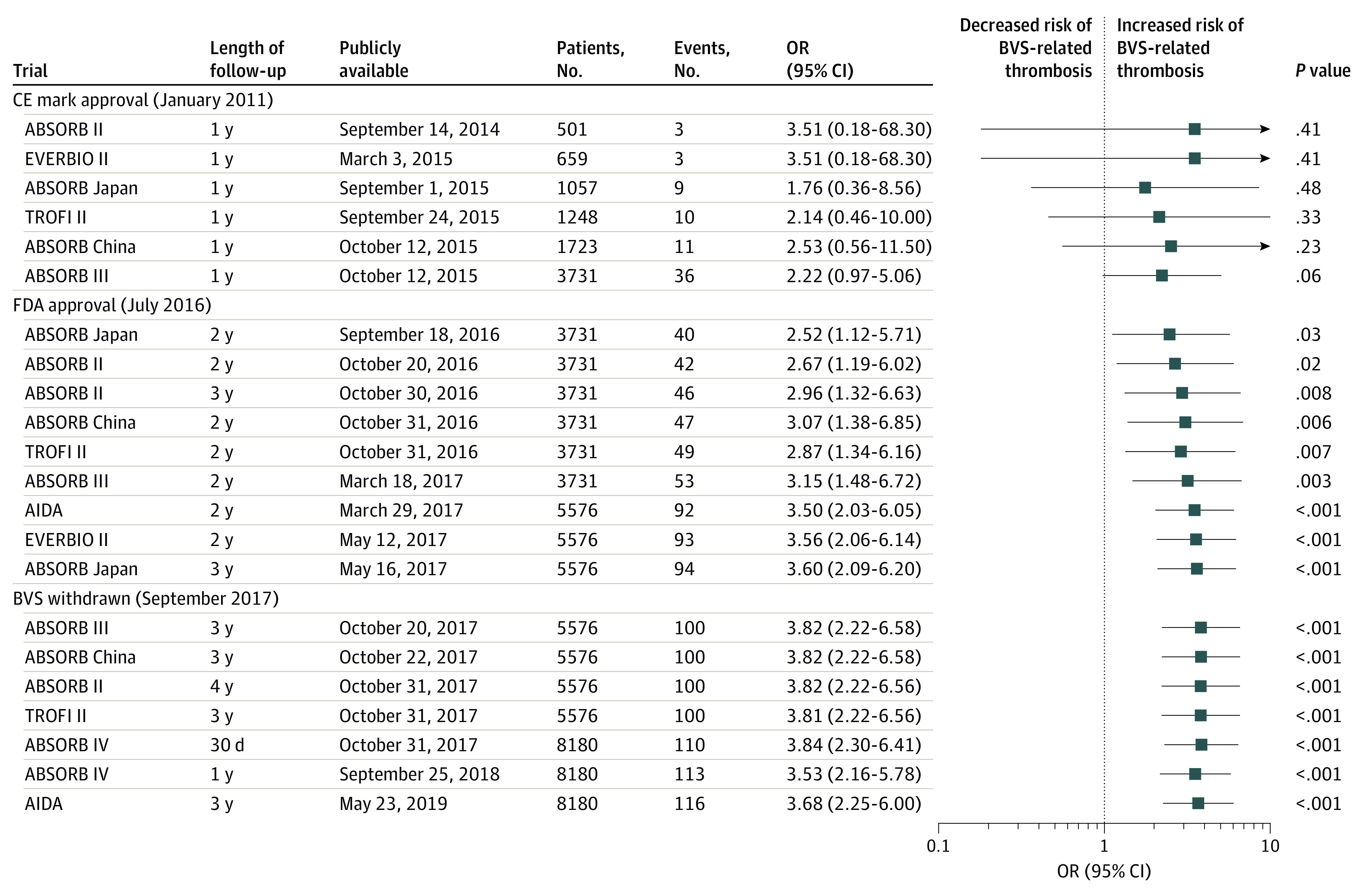
Cumulative Meta-analysis of Trials Comparing the Absorb Polymeric Everolimus-Eluting Bioresorbable Vascular Scaffold With the Metallic Everolimus-Eluting Stent Reports describing increasing follow-up durations for the same trial are ranked according to the date of becoming publicly available. To avoid duplicate counts, data from shorter follow-up periods were omitted from the analysis after data from longer follow-up periods became available. Squares indicate odds ratios (ORs), with horizontal lines representing 95% CIs. BVS indicates bioresorbable vascular scaffolds; CE, Conformité Européene; FDA, US Food and Drug Administration.

A total of 22 reports describing 8 RCTs including 8180 patients randomized to BVS (4553 patients) or everolimus-eluting stents (3627 patients) were included, with 96 and 20 device-related thromboses for each intervention, respectively. Patient recruitment took place over 6 years, with considerable overlap of recruitment periods (Figure 1). The cumulative meta-analysis (Figure 2) revealed that the initial uncertainty regarding the treatment effect based on early trials with follow-up to 1 year gained precision through inclusion of additional trials and follow-up time. The analysis of accumulated evidence showed initial safety concerns after the publication of ABSORB III trial on October 12, 2015, for a clinically important but non–statistically significant increase in the risk of device-related thrombosis after use of BVS (OR, 2.22; 95% CI, 0.97-5.06, *P* = .06). The between-group difference became statistically significant on September 18, 2016 (OR, 2.52; 95% CI, 1.12-5.71; *P* = .03). Availability of longer follow-up and new trials resulted in an OR of 2.87 (95% CI, 1.34-6.16; *P* = .007) 11 months before the Absorb BVS was withdrawn in September 2017. Between-group differences reached on March 18, 2017, had an OR of 3.15 (95% CI, 1.48-6.72; *P* = .003) with a lower limit of the 95% CI above 1.00 (Figure 2). The final estimate was an OR of BVS-related thrombosis of 3.68 (95% CI, 2.25-6.00; *P* *<* .001), indicating that the experimental intervention was harmful.

## Discussion

Timely recognition of safety signals is important to patients, physicians, regulators, and the medical community at large to avoid unnecessary, clinically important adverse events and to prevent waste of research efforts, especially in studies of the comparative effectiveness of medical devices. In the absence of large clinical trials, some adverse events may not be known a priori when a new device is used and additional mechanisms, such as regulatory oversight for unexpected events, may be needed; continuously updated cumulative meta-analyses may contribute to this purpose. Of note, although cumulative statistical testing can bias this approach, it is not of particular concern in the present analysis because it was performed retrospectively and was not associated with a stopping rule for the meta-analysis. However, in a prospectively designed cumulative meta-analysis, correction for multiple testing should be considered because the examination of multiple outcomes and repeated analysis of the data over time may exacerbate the risks associated with multiplicity and further adjustments may be warranted.^3,4^ Under these scenarios, false-positive rates for significance tests at the conventional *P* < .05 are typically too high, and naive interpretations of statistical significance should be avoided.^5,6^
